# Synthesis and characterization of a series of conducting polymers based on indole and carbazole

**DOI:** 10.55730/1300-0527.3471

**Published:** 2022-08-10

**Authors:** Mehmet ERGİNER, Belkıs USTAMEHMETOĞLU, Esma SEZER

**Affiliations:** Department of Chemistry, İstanbul Technical University, İstanbul, Turkey

**Keywords:** Carbazolylindoles comonomers, synthesis, electropolymerization, electronic and optical properties

## Abstract

A series of indole (In) and carbazole (Cz) derivative monomers have been synthesized, such as 4-[3-carbazolyl] indole (4In-3Cz), 5-[3-carbazolyl] indole (5In-3Cz), 6-[3-carbazolyl] indole (6In-3Cz), 7-[3-carbazolyl] indole (7In-3Cz). The comonomers synthesized by Stille coupling reaction were characterized by ^1^H-NMR and elemental analysis. Potentiodynamic method was used for electropolymerization of comonomers, Indole, Cz, and the mixture of In and Cz. Electrochemical activities of resulting P[4In-3Cz], P[5In-3Cz], P[6In-3Cz], P[7In-3Cz], polyindole (PIn), polycarbazole (PCz) and P[In-co-Cz] films were investigated comparatively by CV at different scan rates, electrochemical impedance spectroscopy (EIS) and spectroelectrochemical measurements. The ionization potentials, I_p_, specific capacitance, C_sp_, and optical band gap, E_g_, of copolymers were obtained from these measurements. In order to gain some preliminary information on the structure of the copolymers, DFT analysis was performed and dimers and tetramers were optimized.

Results suggested that, in order to obtain an In-Cz copolymer with low oxidation potential and band gap, indole ring should be substituted through 5 position to the 3 position of Cz. If high specific capacitance value or high conductivity are desired, P[4In-3Cz] and P[6In-3Cz] are the best copolymers, respectively.

## 1. Introduction

There has been a great deal of interest recently in the development of organic-based materials for the applications in the electronics industry. Although less studied than other families of conducting polymers, PCz derivatives could be used in electroluminescent devices as a hole-transporting material, in field effect transistors, batteries, as biosensor, as a light emitting material or a wide band gap energy transfer donor and for other applications [1–3]. Relatively stable radical cations (holes) those easily forms, relatively high charge carrier mobilities, possibility of easy introduction to different substituents, high thermal and photochemical stability, being a cheap raw material, which is readily available from coal tar distillation makes PCz favorable for such applications.

Although PIn has lower electroactivity than other conducting polymers, in recent years due to its important potential in various areas, such as batteries, electronics, sensors, and corrosion protection, it has attracted a significant amount of attention [4–10].

To improve the properties of PIn, electrochemical copolymerization with pyrrole [11,12], thiophene [13] and derivatives [14] and carbazole [15–17] has been explored in the literature. The results showed that these new copolymer materials can offer improved electrochemical activity and stability and better mechanical properties, thereby increasing the application possibilities [18–22].

Another method of obtaining copolymers with desired repeating unit is coupling reactions. While there are a lot of studies on the synthesis the indolocarbazole alkaloids that have indolo[2,3-*a*] Cz as a structure [23–25], studies on substitution of carbazole to benzene ring of indole are less common although it might be electrochemical activity that makes it a good candidate for various applications.

Synthetic methods such as Stille, Yamamoto, Kumada, and Suzuki couplings that led to obtain alternative copolymers with desired properties and improved performance were utilized recently [26–41] and structure-property relationships of result materials can be understood better. Copolymers obtained by these methods exhibit very low optical band gap and switch between different colors, which is potentially useful for electrochromic devices [42]. In the previous work some indoles have been prepared in one step by adding suitable organometallic reagents, followed by LiAlH_4_ to a refluxing mixture of isatin in ether [43]. However, in this study, indole was substituted through the 2-position which limits the polymerization possibilities of indole ring.

In the literature, there are some studies on the synthesis of heteroaryl and allylindoles by Stille cross-coupling reactions [44] that offer advantages to the Suzuki reactions in view of the neutral conditions and the stability and accessibility of the tin derivatives.

Previously, we have also synthesized 5-(2-thienyl) indole (5In-2Th) comonomer [39] and Th-, Cz-, and thiazole-based monomers by using Stille cross coupling reaction [37, 40, 41].

Although there have been previous studies on the synthesis of indole and Cz comonomers with other monomers, studies on the synthesis of In-Cz comonomers are outside of our knowledge. In this study, 4In-3Cz, 5In-3Cz, 6In-3Cz, and 7In-3Cz were synthesized, polymerized, and comparatively characterized for the first time. Their physical properties were obtained by spectroscopic and electrochemical techniques. The results showed that P[4In-3Cz], P[5In-3Cz], P[6In-3Cz], P[7In-3Cz] are promising for applications as they have lower oxidation potentials and optical band gaps than homopolymers.

## 2. Experimental

### 2.1. Materials

Analytical grade chemicals of the highest purity, such as acetonitrile (ACN) and dichloromethane (CH_2_Cl_2_), sodium perchlorate (NaClO_4_), In, Cz, tetramethyl ethylene diamine (TEMED), trimethyl tin chloride (Sn(CH_3_)_3_Cl), MgSO_4_, trans-dichloro bis-triphenyl phosphine palladium (II) (Pd(PPh_3_)_2_Cl_2_), *tert*-buthyl lithium (*t*-BuLi), , K_2_CO_3_, 4-chloro-indole (4-ClIn), 5-bromo-indole (5-BrIn), 6-chloro-indole (6-ClIn), 7-chloro-indole (7-ClIn), were used as supplied. Toluene and tetrahydrofuran (THF) were dried over sodium before use. All preparations and reactions were performed by using standard Schlenk line techniques under a N_2_ atmosphere.

## 3. Measurements

NMR spectra were collected on a Brucker 250 MHz spectrometer and referenced to the residual proton solvent resonance. CHNS-932 LECO model device was used for elemental analysis.

ATR-FTIR measurements were performed with Perkin Elmer spectrum one spectrophotometer.

Keithley 2400 model multimeter connected to Lucas labs 302 model probe holder and SP4-180-TFS type probe was used to measure solid state electrical conductivity of polymers from pellets with a thickness of 0.8 mm. The following equation was used for calculation [45, 46]:


$$\sigma ={\text{V}}^{-1}\;\text{I }(\text{ln }2/\pi \;{\text{d}}_{\text{n}})$$

where σ = conductivity, V is the potential in volts, I is current in ampere, and d_n_ is the thickness in cm which was measured by using a digital caliper.

PARSTAT 2263 model potentiostat/galvanostat controlled with Power Suite software via a computer was used for electrochemical polymerization and characterization. Measurements were performed in a three-electrode cell that contains a Pt button (area of 0.02 cm^2^), Pt wire, and Ag wire as the working, axillary, and reference electrode, respectively. Calibration of pseudo-reference was performed externally by using a 5 mM ferrocene/ferrocenium solution that has a potential 0.35 V vs. Ag/AgCl. Spectroelectrochemical measurements were performed by Shimadzu 160A model UV-visible spectrophotometer in a quartz cell. Indium tin oxide (ITO) coated glass electrode with a size of 7 cm **×** 5 cm **×** 011 cm that has a resistivity R ≤12 Ω/cm obtained from Colorado Concept Coatings Company was used as working. Pt wire and Ag wire were counter and a reference electrode, respectively. Capacitive behavior of polymers was tested by electrochemical impedance spectroscopy (EIS) measurements that performed in the range of 10 **×** 10^3^Hz–10 **×** 10^−3^ Hz with 10 mV amplitude at open circuit potential.

Density functional theory (DFT) method at 6–31g* level was used for optimization of comonomer and oligomer geometries. Gaussian software [47] was used for the calculations on the supercomputer of High-Performance Computing Center at ITU.

## 4. Synthesis

In-Cz comonomers were synthesized by Stille coupling reactions between haloindoles and 3-trimethyl tin Cz which was synthesized by the reaction of Cz and Sn (CH_3_)_3_Cl.

### i) Synthesis of 3-(trimethyl stannyl)carbazole

Firstly, a 100-mL Schlenk flask equipped with a stirring bar and N_2_ inlet/outlet was dried and charged with ~80 mL of THF, 2.00 g (12 mmol) of Cz, and 1.39 g (12 mmol) of TEMED. After cooling the flask to −78 **°C**, 2.5 M (12 mmol) *t*-BuLi in hexanes was added and stirred at this temperature for 1 h. Then, 2.39 g (12 mmol) of Sn (CH_3_)_3_Cl in 15 mL of THF was added dropwise. The mixture was stirred overnight at room temperature. After evaporation of solvent under vacuum, 100 mL of CHCl_3_ was added to the mixture. The extraction of product from solution was performed by CHCl_3_ and washing with water. Organic phase was collected dried by MgSO_4_. The solvent was evaporated under vacuum and a dark yellow product was obtained. The product was characterized with ^1^H NMR and FT-IR measurements.

(Yield = 1.3 g, 74%). ^1^H NMR (in CDCl_3_): δ (ppm) (8.1, 2H, dd), 8.0 (1H, s), 7.6–7.4 (4H, m), 7.2 (1H, s), 0.34 (9 H, s)

In the FT-IR spectrum, the presence of –N-H str. at 3412 cm^−1^, aliphatic –C - H str. of trimethyl group at 1935–1695 cm^−1^, –C = C aromatic ring str. at 1625–1411 cm^−1^, –C - Sn str. at 1090–996 cm^−1^, –C - Sn out of plane deformation at 798 cm^−1^, supported the synthesized Sn-Cz structure.

### ii) Synthesis of carbazolyl-indole comonomers

Firstly, 7.65 mmol (2.86 g) 4-bromo indole, 7.65 mmol (1.5 g) 3-trimethyl stannyl carbazole, and 0.3 mmol (0.012 g) trans-dichloro bis triphenylphosphine palladium (II) chloride in 100 mL of toluene solution was prepared and reaction was continued for 24 h under reflux.

At the end of the reaction, mixture was poured into water and extracted with CH_2_Cl_2_. The process was repeated three times and the organic phases were combined and dried with K_2_CO_3_. After evaporation of some solvent, purification of the product was carried out by flash chromatography that contains silica gel as a stationary phase and CHCl_3_/hexane/THF; 1:1:1, v/v mixture as eluent.

**Scheme 1.**
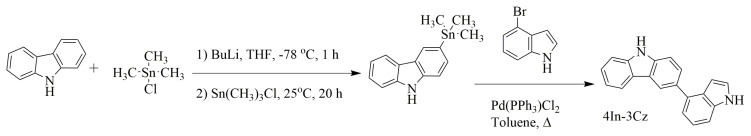
Synthesis of 3-(trimethyl stannyl) carbazole and 5-[3-carbazolyl] indole.

**Scheme 2.**
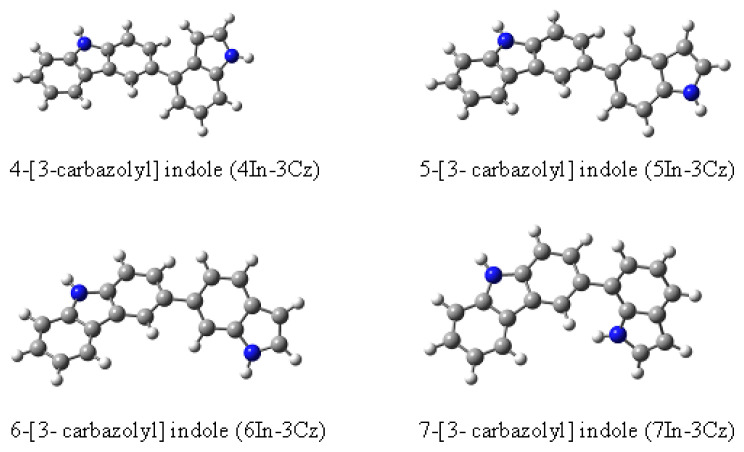
Structure of In-Cz comonomers.

Synthetic route and the structures of synthesized comonomers are shown in Schemes 1 and 2, respectively.

**Figure 1:**
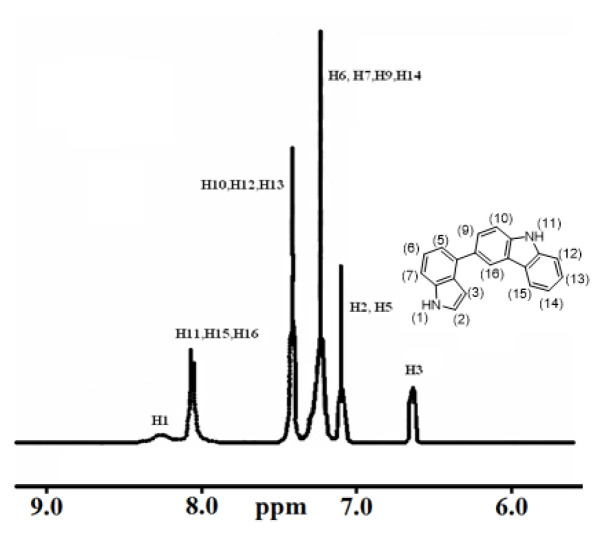
^1^H-NMR Spectrum of 4In-3Cz. Protons of 4In-3Cz comonomer **(inset)**.

Comonomers were fully characterized by ^1^H-NMR and elemental analyses and results are given below. Observation of all numbered protons (inset) of 4In-3Cz comonomer, as an example, given in ^1^H-NMR spectrum, supported the synthesis of comonomers (Figure 1).

The yields and the elemental analysis of the comonomers are given below:

**4In-3Cz** Yield: %50. Elemental analysis: C: 85.6%, H:6.3%, and N:7.9%. H1-NMR: (250 MHz, in CDCl_3_) (δ, ppm); 6.45 (H3) 7.1–7.00 (H2, H5), 7.20–7.30 (H6, H7, H9, H14), 7.4–7.5 (H10, H12, H13), 8.05 (H11, H15, H16), 8.15 (H1).

**5In-3Cz** Yield: 40%. Elemental analysis: C: 85.12%, H: 4.96%, N: 9.3%. H1-NMR: (250 MHz, in CDCl_3_): (δ, ppm); 6.50 (H3) 7.0–7.25 (H2, H6, H7, H14), 7.4–7.5 (H9, H10, H12, H13), 7.6(H4), 7.81 (H1), 8.03 (H11), 8.10 (H15, H16).

**6In-3Cz** Yield: 47%. Elemental analysis: C: 84.74%, H: 5.9%, N: 7.871%. H1-NMR: (250 MHz, in CDCl_3_) (δ, ppm); 6.52 (H3), 7.05(H2), 7.12(H5), 7.24(H14), 7.27(H7), 7.40–7.45 (H9, H10, H12, H13), 7,64 ( H4), 7.83 ( H1), 8.05 (H11), 8.16 ( H15, H16).

**7In-3Cz** Yield: 57%. Elemental Analysis: C: 85.47%, H: 5.03%, N: 8.82%. H1-NMR: (250 MHz, in CDCl_3_): (δ, ppm); 6.47 (H3), 7.05(H2), 7.12(H5), 7.18(H6), 7.24(H14), 7.42–7.48( H9, H10, H12, H13), 7,64 ( H4), 7.81 (H1), 8.04 (H11), 8.09 (H15, H16).

Elemental analysis results of comonomers agreed with theoretical ones; C: 85.71 %, H: 4.29 %, N: 10.00 %. These results suggested that the comonomer structure contains one Cz unit for one indole unit.

### 4.1. Electropolymerization

The electropolymerization was performed in 0.1 M NaClO_4_ containing ACN on Pt by potentiodynamic method in the potential range of −0.2 V (vs. SCE) up to 1.8 V at 20 mV s^−1^. Different cycle numbers were applied during electropolymerization to obtain polymer films at different thicknesses and optimum thicknesses were determined according to the redox behaviors.

For comparison, random copolymer of indole and Cz was also prepared starting from their mixtures under the same conditions and called P[In-co-Cz]. The exact concentrations of pristine monomers (Cz, In, and In-Cz comonomer) were 1.0 **×** 10^−3^ M and the total concentration in the mixture of In and Cz was also 1.0 **×** 10^−3^ M by using 0.5 **×** 10^−3^M In and 0.5 **×** 10^−3^M Cz.

All polymeric films were washed with ACN before characterization.

### 4.2. Voltametric measurements

**Figure 2:**
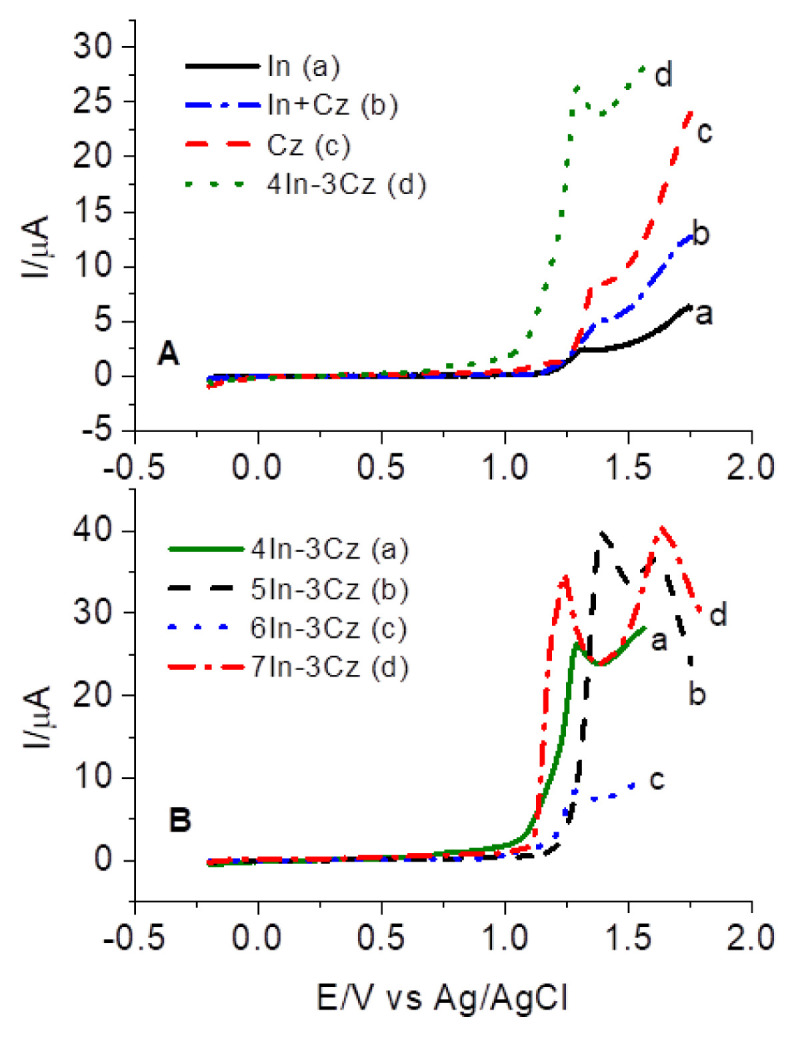
Anodic polarization curves **a)** In, **b)** In+Cz mixture, **c)** Cz, **d)** 4In-3Cz. **(A)** and **a)** 4In-3Cz, **b)** 5In-3Cz, **c)** 6In-3Cz, **d)** 7In-3Cz **(B)** in ACN containing 0.1 M NaClO_4_.

Electrochemical oxidation onset potentials (E_ox_) from CVs were obtained at the position where the current starts to increase. Figure 2A shows the first anodic scan of CVs for In, Cz, In + Cz mixture, and 4In-3Cz and their E_ox_ values were determined from this figure as 1.14, 1.24, 1.16, and 1.06 V, respectively. The mixture of In and Cz has a current value that is between what In and Cz individually have, as expected. A solution containing 1.75 × 10^−4^ M 4In-3Cz showed lower E_ox_ than the others and the current value was the highest. This result suggested that the 4In-3Cz behaved differently than the mixture of In and Cz, which supported the formation of comonomer. When the polarization curves of comonomers were compared, it can be seen that the E_ox_ values were close to each other and current intensities at maximum peak potentials were shifted and 6In-3Cz has the lowest value (Figure 2B).

Ionization potential (I_p_) was calculated as suggested in the literature [48]:


(1) $${\text{I}}_{\text{p}}={\text{E}}_{\text{ox}}+4.4\;(\text{eV})$$

E_ox_ and calculated I_p_ values of monomers are summarized in Table 1.

The results suggested that it is possible to obtain new comonomers by cross-coupling reactions of Cz and In with lower oxidation potential than the value of starting monomers.

**Figure 3:**
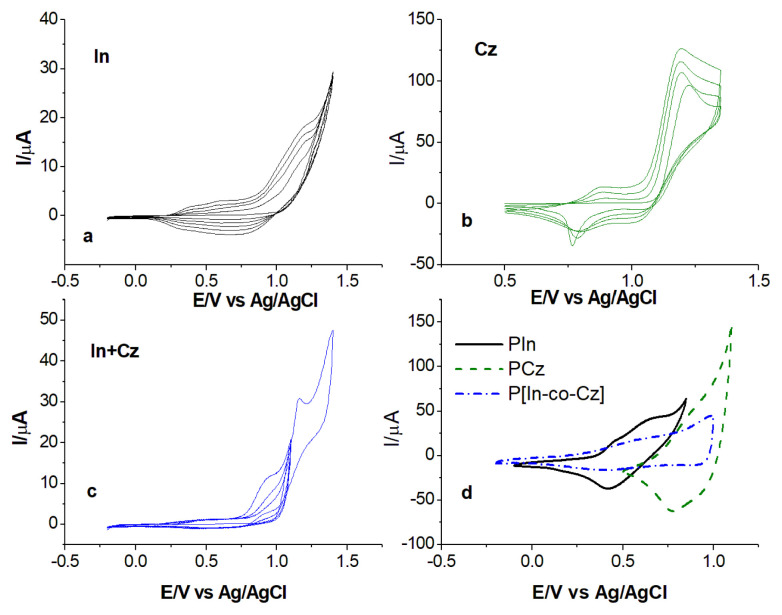
CVs of 1.0 × 10^−3^ M In **(a)**, 1.0 × 10^−3^ M Cz (**b)**, and 0.5 × 10^−3^ In + 0.5 × 10^−3^ M Cz mixture (**c)** at 20 mV/s and CV of PIn, PCz and P[In-co-Cz] at scan rate of 200 mV/s **(d)** in an ACN/0.1 M NaClO_4_ solution.

In, Cz, and In + Cz were polymerized potentiodynamically, and electroactivities of the polymers were tested by CV performed at variety of scan rates and results are illustrated in Figure 3.

Oxidation started at 1.14 V, 1.24 V, and 1.16 in the first scan of In, Cz, and In + Cz, respectively, which were attributed to formation of cation radicals by oxidation of the monomers (Figures 3a–3c). In the first cycle, when potential up to 1.4 V is applied for In + Cz similarly with the polymerization of In and Cz, a faster current increase was observed after the peak potential of around 1.1 V. For this reason, the applied potential has been reduced to prevent overoxidation of the copolymer film formed on the surface, which has lower redox activity (Figure 3c). In the following cycles, a new peak appeared at lower potentials, 0.62 V (E_P1_), 0.87 V (E_P1_) and 0.52 V (E_P1_), 0.93 V (E_P2_) for In, Cz, and In + Cz respectively. The peak current increased upon successive cycling and it was an indication of the deposition of polymer.

The redox activity of the polymers was tested by CV in ACN containing 0.1 M NaClO_4_ at different scan rates. Comparison of the CVs of resulted polymers namely, PIn, PCz, and P[In-co-Cz] is given in Figure 3d. P[In-co-Cz] has a peak potential in between PIn and PCz, as expected.

**Figure 4:**
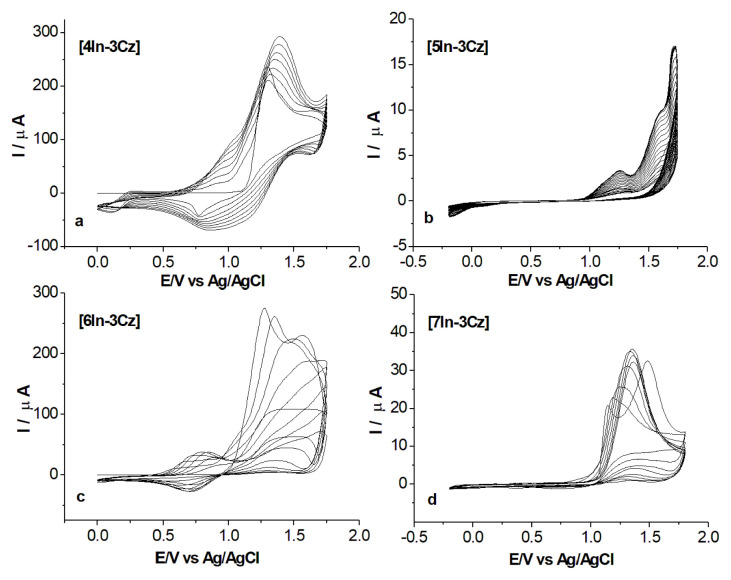
CV of 1.0 × 10^−3^ M 4In-3Cz **(a)**, 5In-3Cz **(b)**, 6In-3Cz **(c)** and 7In-3Cz **(d)** in ACN containing 0.1 M NaClO_4_ solution with at a scan rate of 20 mV/s.

During electropolymerization of In-Cz comonomers, CVs were obtained and are given in Figure 4. The anodic potential limit of polymerization in all cases was selected as 1.75 V, since 4In-3Cz, comonomer does not polymerize at lower potentials. In order to optimize the polymerization conditions, the same potential range was selected for all monomers. Increase in current intensity was higher in the case of 4In-3Cz and 6In-3Cz than 5In-3Cz and 7In-3Cz and this result suggested that thicker film formation occurred in the case of 4In-3Cz and 6In-3Cz comonomers.

**Figure 5:**
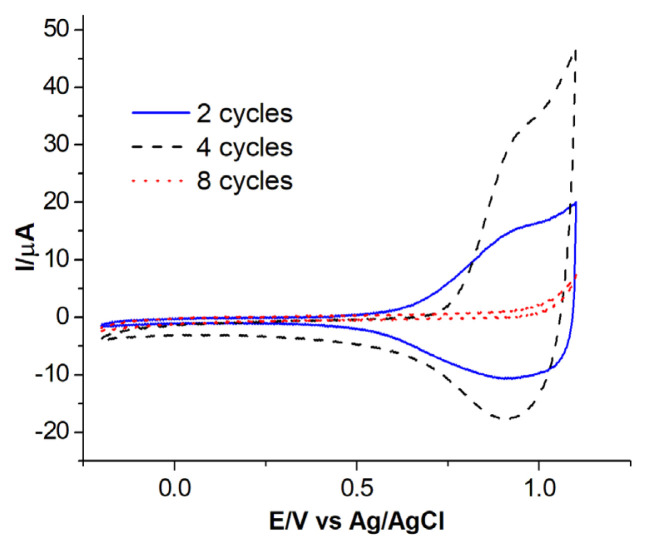
Comparison of cyclic voltammograms of P[7In-3Cz] films obtained by potentiodynamic method by applying 2, 4, and 8 cycles, in ACN containing 0.1 M NaClO_4_. Scan rate: 200 mV/s

The redox behaviors of resulting polymeric films obtained by applying different cycle numbers were compared (Figure 5). For example, for the P[7In-3Cz], the optimum number of cycles was accepted as 4, since the redox behavior of the film obtained by applying 4 cycles was better than the film obtained in 2 and 8 cycles.

**Figure 6:**
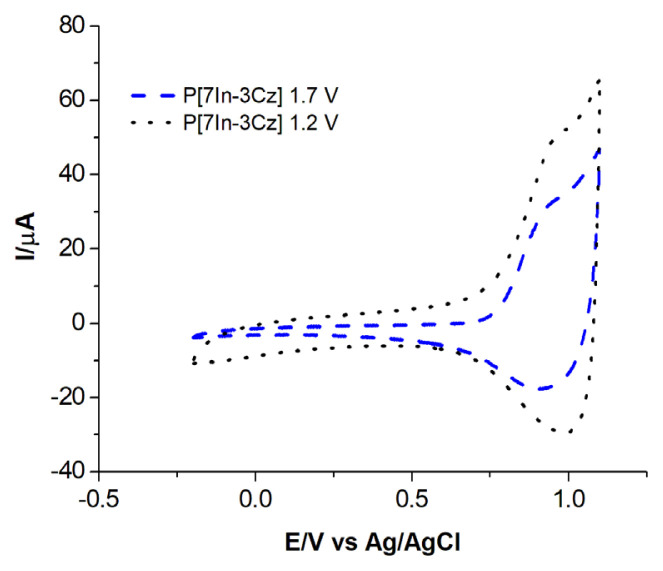
CV of P[7In-3Cz] film obtained potentiodynamically at two different potential ranges (0.0–1.2 V and 0.0–1.7 V).

Polymerizations were also carried out in different potential ranges for 5In-3Cz, 6In-3Cz, 7In-3Cz. It was found that the redox behavior of polymer depends on the anodic potential limit and at lower potential limit; more electrochemically reversible polymeric films were obtained as expected [49]. This comparison was given for P[7In-3Cz] as an example (Figure 6). As it can be seen the polymer film obtained at the potential limit of 1.75 V has quasireversible redox behavior due to some degradation in the polymer chain.

**Figure 7:**
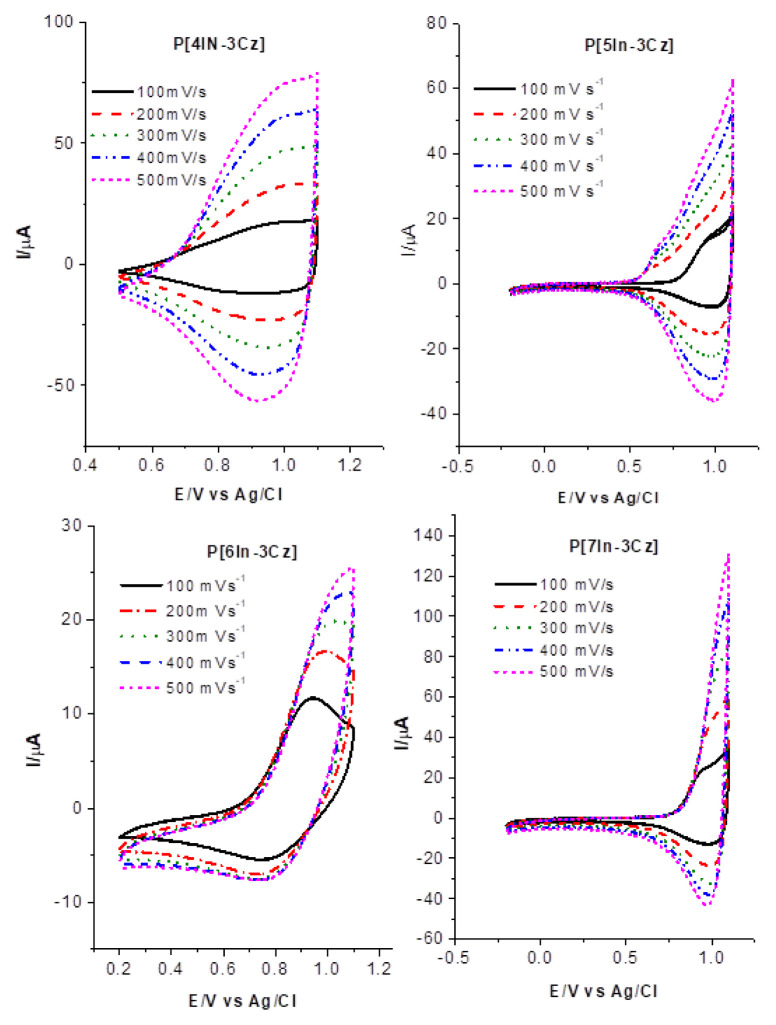
CV of a P[4In-3Cz] **(a)**, P[5In-3Cz] **(b)**, P[6In-3Cz] **(c)** and P[7In-3Cz] **(d)** ACN containing 0.1 M NaClO_4_ at different scan rates.

Scan rate dependence of P[In-Cz]’s is given in Figure 7.

De-doping peak of P[6In-3Cz] was not significant as much as the peaks of P[4In-3Cz], P[5In-3Cz] and P[7In-3Cz]. Generally, CV of conducting polymers demonstrates “irreversible” anodic and cathodic peaks and there are several explanations for such behaviors [50]. Free rotation of the molecule during the transition from benzonoid to quinoid structure might be hindered by deposited oligomers on the electrode as a solid matrix and the typical voltammogram is the superposition of different redox waves, i.e. a mixture of oligomers, and polymers of different chain lengths and possibly different cross-linking. Role of coupling position seems to affect the redox behavior of polymer and superposition of different redox waves became more pronounced at lower scan rates for P[5In-3Cz] and P[7In-3Cz].

**Figure 8:**
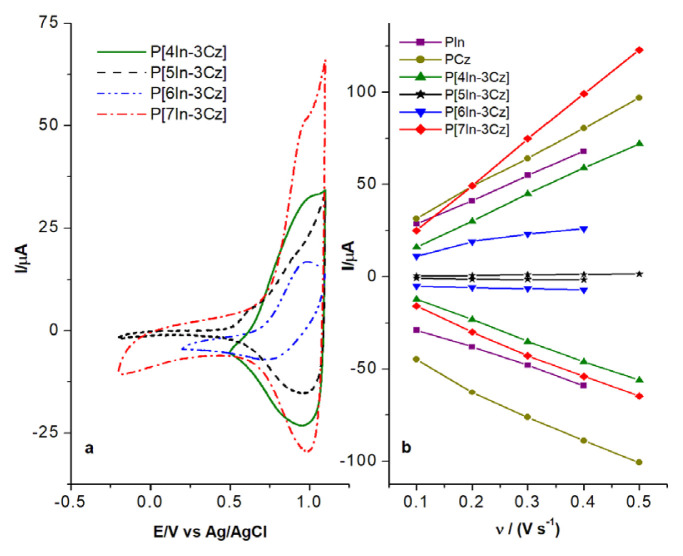
CV of P[4In-3Cz], P[5In-3Cz], P[6In-3Cz], P[7In-3Cz] at a scan rate of 100 mV/s **(a)** and scan rate dependence of the anodic and cathodic peak currents **(b**) of polymer films obtained on the Pt electrode.

The comparison of CVs of all In-Cz polymer films is given in Figure 8a. As it can be seen, although the peak potentials of polymers were close to each other, the highest peak current was observed for P[7In-3Cz]. This result indicated that the coupling position affected the electroactivity of P[In-Cz] films and P[7In-3Cz] seems to be the most electroactive one.

Scan rate dependence on the anodic and cathodic peak currents (I_pa_ and I_pc_) of P[4In-3Cz], P[5In-3Cz], P[6In-3Cz] and P[7In-3Cz] was investigated comparatively with PIn and PCz obtained under the same experimental condition. It was found that I_pa_ and I_pc_ scale linearly with scan rates not with square root of scan rates and this result indicate an electroactive thin film behavior instead of diffusion control one as suggested in literature [49] (Figure 8b). The order of the current intensities was found as follows: P[7In-3Cz] > PCz > PIn > P[4In-3Cz] > P[6In-3Cz] > P[5In-3Cz].

### 4.3. FT-IR results

**Figure 9:**
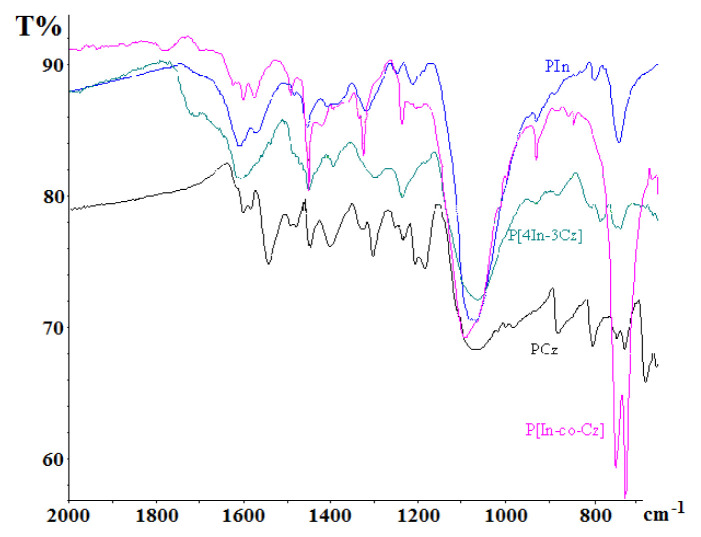
ATR-FTIR spectra of PIn, PCz, P[In-co-Cz] and P[4In-3Cz].

The FT-IR spectra of PIn and PCz, P[In-co-Cz]) and P[4In-3Cz] are given in Figure 9. The characteristic vibrations of Cz and indole rings are very similar. Therefore, it is difficult to interpret the coupling position. Two rings give similar vibration at very close wave numbers and when they coupled each other these peaks are split. –C - H out of plane deformation peaks were observed at 739 and 794 cm^−1^ for PIn, and at 727, 746, and 800 cm^−1^ for PCz and split into three peaks in the range of 810–734 cm^−1^ in the case of P[4In-3Cz]. This result showed the presence of both In and Cz in the structure of P[4In-3Cz]. The peak at 1400 cm^−1^, which belongs to the aromatic -C - H vibration, was observed in PCz, but not in PIn, and shifted to 1393 cm^−1^ in the case of P[4In-3Cz]. This result indicates that Cz is included in the structure. Similarly, the presence of the peak at 927 cm^−1^ for PIn but the absence for PCz, observed at 927 cm^−1^ for P[4In-3Cz] showed the inclusion of indole to the P[4In-3Cz] structure. An absorption band at 1100 cm^−1^ which is attributed to ClO_4_^−^, showed doping of the polymers with this anion (Figure 9). The peak at 740 cm^−1^ corresponding to the out of plane deformation of aromatic –C - H of Cz and indole, was observed with higher intensity for P[In-co-Cz] by splitting in two (745 and 720 cm^−1^). This indicated less coupling possibility through the benzene ring during the random copolymerization of In and Cz.

### 4.4. Optical properties

**Figure 10:**
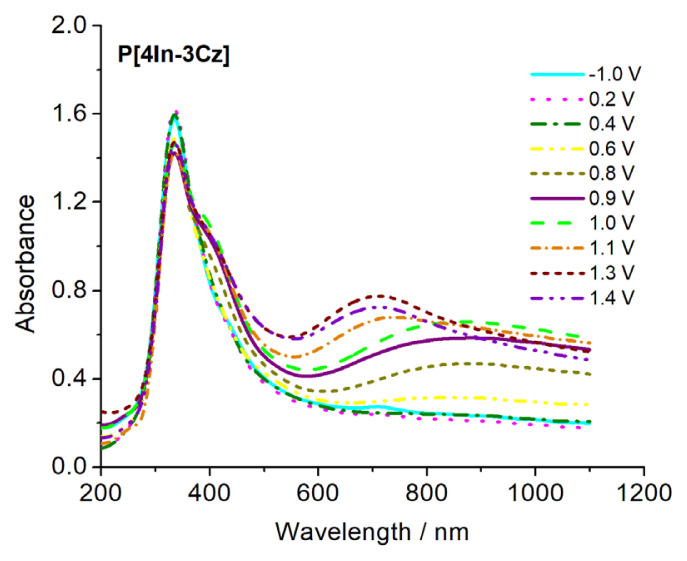
UV-visible absorption spectra of P[4In-3Cz].

Spectroelectrochemical methods were also employed to study optical properties of polymers by applying anodic potentials which results polaron and bipolaron formations. P[4In-3Cz], P[5In-3Cz], P[6In-3Cz] and P[7In-3Cz] films were electrochemically polymerized on ITO for spectroelectrochemical analysis and subsequently placed in a quartz cuvette with Pt counter and Ag reference electrodes. For reduction and to obtain their neutral states, −1.0 V was applied to the polymers. UV-visible spectrum of P[4In-3Cz] obtained by applying different potential is shown in Figure 10. The peak at 350 nm was observed it was attributed to π-π* transition. The new peak at 870 nm was started to form by application of anodic potential at 0.8 V and this might be due to polaron and/or bipolaron formation. As the anodic potential was increased (up to 1.1 V and further), absorption maximum shifted to 700 nm. This might occur by oxidation of polymer chains that results better and less ordered polymer structures as suggested for other PTh derivatives [51–53]. Another explanation might be formation of the segments with different conjugation length [54] and these two approaches are very close to each other.

E_g_ values of polymers were estimated from UV-visible spectrophotometric measurements, by extrapolation of the low energy edge to the baseline of the spectra and results are given in Table 2. While P[4In-3Cz] and P[6In-3Cz] have lower E_g_ than PIn and PCz, P[5In-3Cz] has similar value with Pin, and P[7In-3Cz] has the highest E_g_. It is found that E_g_ could be decreased by obtaining polymers from In-Cz comonomer that synthesized by coupling of In and Cz instead of random copolymerization.

E_ox_ of P[In-Cz]s were calculated from the equation (1) above and showed similar trends with comonomers. Results are given in Table 2. It can be seen that E_ox_ of P[In-Cz]s was slightly different from the values of PIn and PCz. E_ox_ and E_g_ values of conjugated polymers are expected to decrease with the extension of π-conjugation length. When the E_ox_ of polymers and monomers are compared (Table 2), it can be seen that E_ox_ significantly decreases in the case of polymers. Variation in molecular architecture of resulting polymer has different effects on ionization potential and electron transition between the HOMO and LUMO levels; therefore, changes in E_ox_ values were different from variation of E_g_ values.

### 4.5. EIS measurements

**Figure 11:**
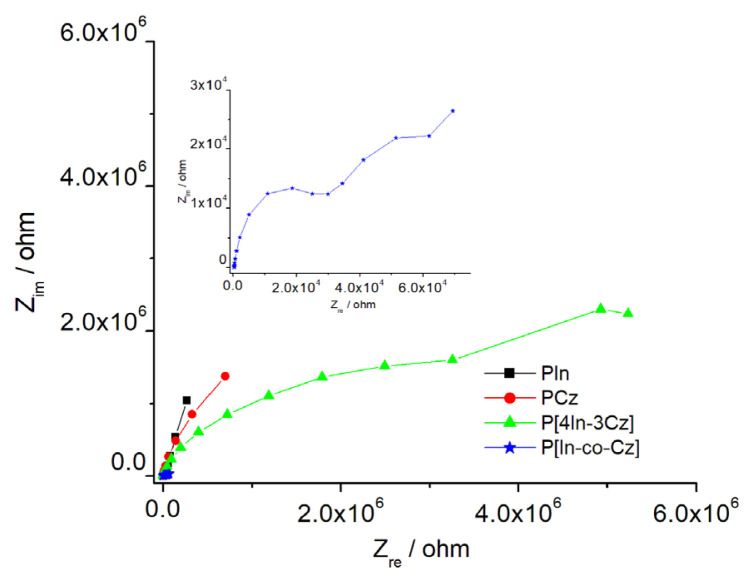
Nyquist diagrams of PIn, PCz, P[In-co-Cz], and P[4In-3Cz] films in the frequency range of 10 × 10^3^Hz–10 × 10^−3^ Hz at open circuit potential. Enlarged Nyquist diagram of P[In-co-Cz] at high frequency region **(inset)**.

To explore the capacitive behavior of polymers, EIS measurements were performed and Nyquist diagrams of PIn, PCz, P[In-co-Cz], and P[4In-3Cz] films are given in Figure 11. Specific capacitance values (C_sp_) were calculated from the slope of imaginary component of the impedance, -Z_im_ versus 1/frequency (f) plot, by using the equation below as suggested in the literature [55] and the results are summarized in Table 2.


$$Z_{im}=\frac{1}{2\pi C_{sp}}\frac{1}{f}$$

Capacitive behaviors of polymers depend on the structure, and the highest C_sp_ value was obtained in the case of P[4In-3Cz]. This structure seems to be the most favorable one for charge storage applications. The C_sp_ values of the copolymers needed to be improved by changing reaction conditions for application.

**Figure 12:**
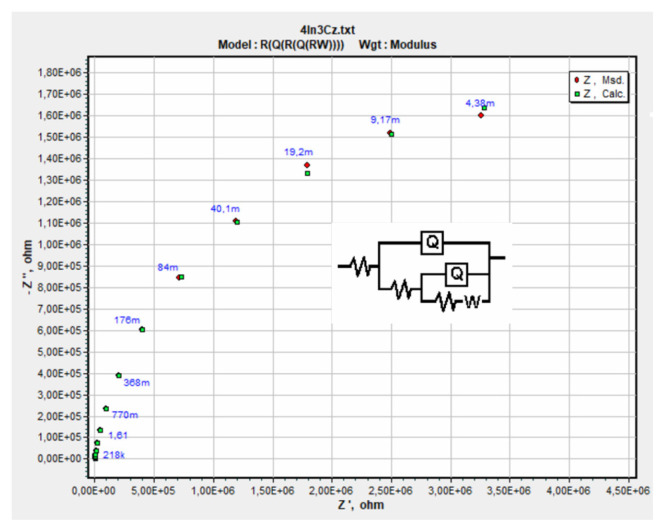
Experimental and simulated Nyquist diagrams of P[4In-3Cz] film with. Equivalent circuit model (**inset**).

Experimental EIS results were analyzed by fitting an equivalent circuit model. Different electrical models were tried and with the one that has the lowest chi-squared value, an excellent agreement between experimental and simulated data was obtained as shown in Figure 12. The values of the circuit elements obtained as a result of the simulation are summarized in the Table 3.

The value of R1 in the circuit belongs to the solution resistance of the cell, constant phase elements (CPEs) and R_2_ and R_3_ belong to the nonideal capacitances and resistances of the polymer film and electrode, respectively, and W to the Warburg element related to mass transfer.

The impedance of a capacitor is obtained by the following equation and the CPE value is used in case of nonideal capacitors account for surface roughness, nonuniform current distribution etc. and the exponent n is less than one:


$${\text{Z}}_{\text{CPE}}=\frac{1}{{\left(\text{j}\omega \right)}^{\text{n}}{\text{Y}}_{0}}$$

where Y_0_ is the capacitance, C, and n is an exponent equaling 1 for ideal capacitor

The equation for Warburg impedance and can be written as follows:


$${\text{Z}}_{\text{w}}=\frac{\frac{1}{{\text{Y}}_{0}}}{\sqrt{\text{j}\omega }}$$

where ${\text{Y}}_{0}=\frac{1}{\sqrt{2}\sigma }$ and $\sigma =\frac{\text{RT}}{{\text{n}}^{2}{\text{F}}^{2}\text{A}\sqrt{2}}\left(\frac{1}{{\text{C}}_{0}\sqrt{{\text{D}}_{0}}}+\frac{1}{{\text{C}}_{\text{R}}\sqrt{{\text{D}}_{\text{R}}}}\right)$

ω = radial frequency (s^−1^)
D_o_ = diffusion coefficient of the oxidant (cm^2^/s)
D_R_ = diffusion coefficient of the reductant (cm^2^/s)
A = surface area of the electrode (cm^2^)
n = number of electrons transferred
C = bulk concentration of the diffusing species (moles/cm^3^)

Total capacitance, C_spEIS_, was calculated and is given in Table 3. When experimentally determined and simulated capacitance values were compared, they were found to be in good agreement.

### 4.5. Conductivity measurements

Electrodeposition leads to a doped and conductive polymer, where the perchlorate ion is the dopant. The solid-state conductivities of the polymer films are given in Table 2.

The conductivity values of the polymers agree with literature and have the expected values in the range for PIn and PCz [1, 56, 62, 63]. The highest conductivity was obtained in the case of P[6In-3Cz]. This could be due to higher conjugation possibility and change in growth behavior of P[6In-3Cz] that affects the inclusion of dopant ion.

### 4.6. Possible dimers and tetramers structures of comonomers

E_ox_, E_g_, conductivity, C_sp_ values of copolymers suggested that different structures were obtained by polymerization of comonomers and the mixture of two monomers. Although only random copolymers can be obtained from the mixtures, it is possible to obtain alternated structures by using newly synthesized comonomers which have improved properties. Theoretical and experimental methods were used together in order to have an idea about the relationship between the application-oriented properties of polymers such as E_ox_, E_g_, and C_sp_.

**Scheme 3.**
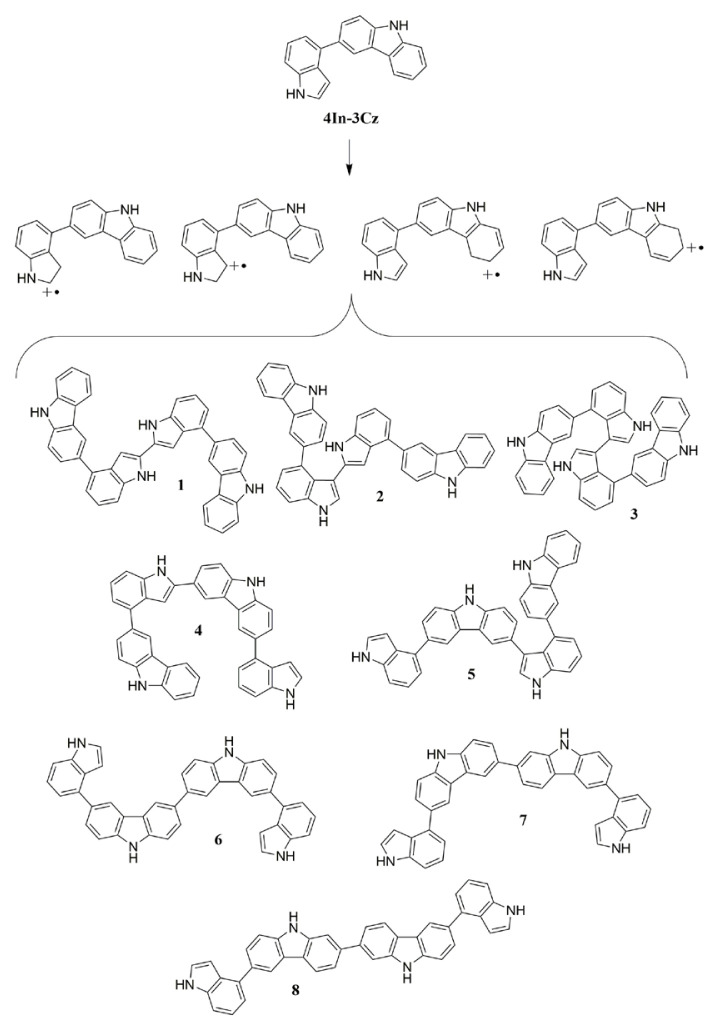
Possible dimers to be formed from the 4In-3Cz comonomer.

The possibility of polymerization from 2 and 3 positions of indole [57–61] and 3 position of Cz [62–64] allows the formation of many dimers for each comonomer as given in Scheme 3 due to asymmetric structure of comonomers. Dimers 7 and 8 are less likely to occur during the polymerization since they coupled through the 2 position of Cz.

As it can be seen, by oxidation of 4In-3Cz comonomer cation radicals can be formed which was coupled in the following step and formed 2,3-indole-indole dimer, called D[2,3(In-In)]_4In-3Cz_. The numbers and abbreviation of the name of dimers that may occur from 4In-3Cz cation radicals are summarized in Table 4.

**Scheme 4.**
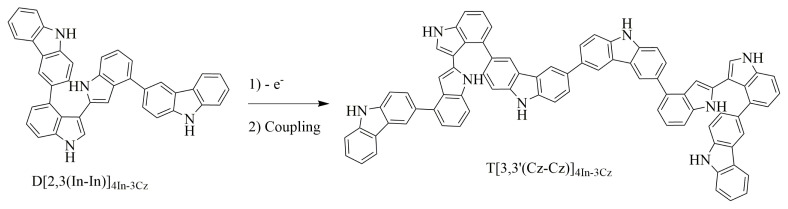
Coupling steps of the formation of D[2,3(In-In)]_4In-3Cz_ dimer and T(3,3′(Cz-Cz)]_4In-3Cz_ tetramer.

In the propagation step, after oxidation, these dimers can couple with each other and formed tetramers, for instance, D[2,3(In-In)]_4In-3Cz_ can couple through 3,3′ position of Cz and give the tetramer named as T(3,3′(Cz-Cz)]_4In-3Cz_ (Scheme 4).

Similarly, other possible dimer structures of 4In-3Cz might be obtained by coupling of Cz cation radicals that formed at the 3 position of Cz rings and called D[3,3′(Cz-Cz)]_4In-3Cz_. Further oxidation of this dimer might result a tetramer by coupling of indole rings through their 2 positions which resulted a tetramer named T[2,2′(In-In)]_4In-3Cz_. Coupling positions have significant role in the properties of resulting copolymers. DFT allowed to resolve the problems concerning the monomer linkage as suggested in the literature for polymerization of 1,8-diaminocarbazole [65]. It would be better to carry out similar calculations to clarify the most probable structures.

**Scheme 5.**
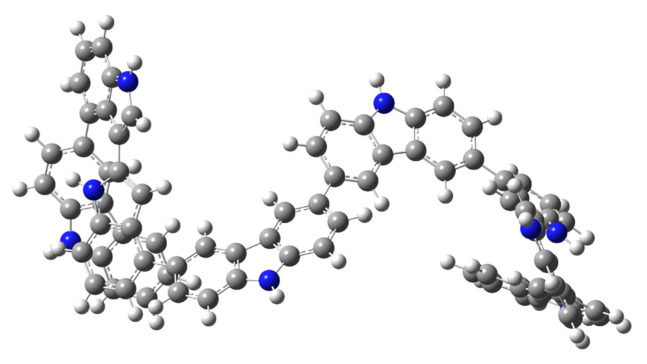
Optimized geometry of T(3,3′(Cz-Cz)]_4In-3Cz_ tetramer formed from D[2,3(In-In)]_4In-3Cz_ dimer.

**Scheme 6.**
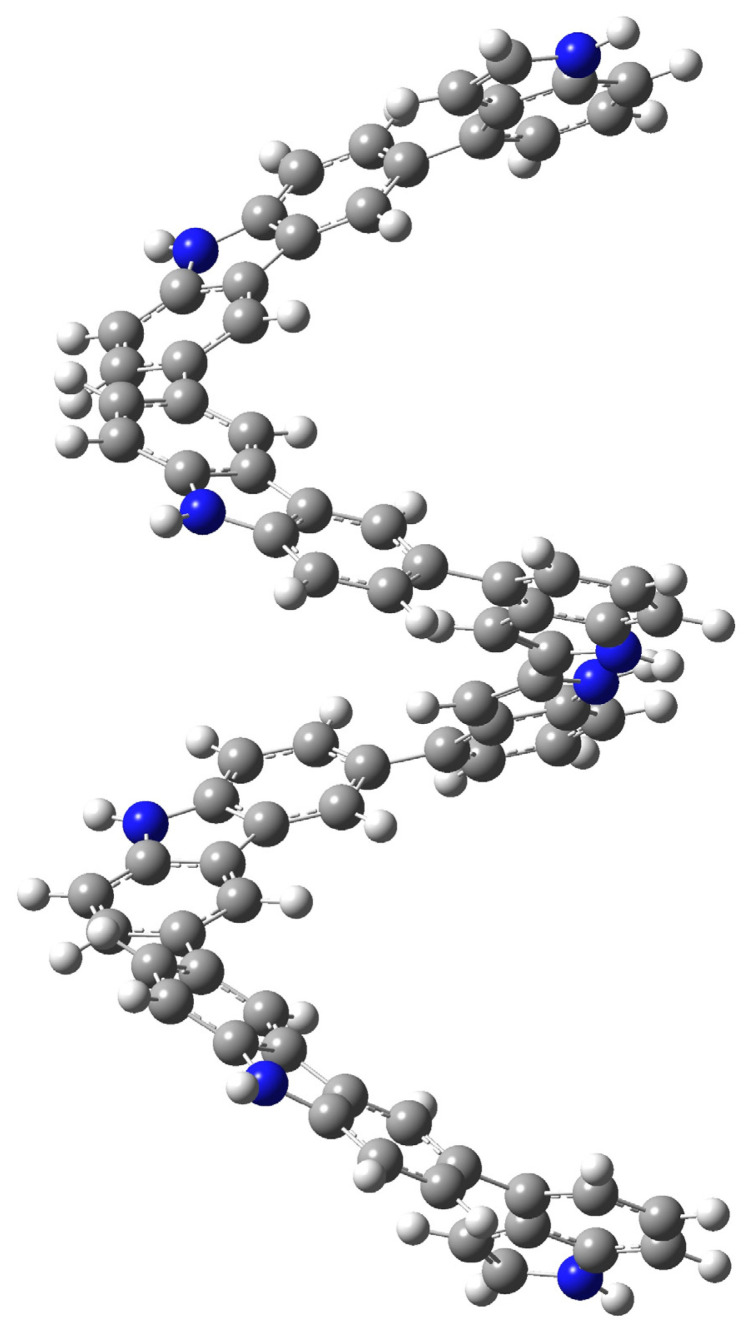
Possible optimized geometry of T[2,2′(In-In)]_4In-3Cz_ tetramer that formed from D[3,3′(Cz-Cz)]_4In-3Cz_ dimer.

Preliminary calculation was carried out for T(3,3′(Cz-Cz)]_4In-3Cz_ and T[2,2′(In-In)]_4In-3Cz_ and optimized geometries of them are given in Schemes 5 and 6. As can be seen, the geometry of the tetramer changes significantly as the coupling position changes, and since the electronic properties of polymers are formed by the contribution of all oligomers, it is difficult to make a precise prediction about the mechanism and structure-property relationship without further theoretical investigation

## 5. Conclusion

The Stille cross-coupling reaction was applied to obtain various carbazolylindoles and their chemical structures were fully characterized by common techniques.

Although random copolymers can be obtained by polymerization of In and Cz mixture, alternated copolymers have been obtained by using newly synthesized In-Cz comonomers.

In + Cz mixture and In-Cz comonomers were electropolymerized and the important properties of their polymers for applications such as redox potential, conductivity, optical band gap and specific capacitance were investigated comparatively.

The changes of optical and electrical properties of In-Cz copolymers synthesized by different indole derivatives showed the role of coupling position of indole ring with Cz. For example, the higher conductivities were obtained in the case of P[6In-3Cz]. Conductivity depends on the conjugation length and geometries of structure. Therefore, it can be said that P[6In-3Cz] has the most planar geometries and/or the highest conjugation length. The evaluation of the relationship between the chemical structure of copolymers and their specific properties were supported by theoretical calculations.

To obtain an In-Cz copolymer with low oxidation potential and band gap, one must substitute indole ring through 5 to the 3 position of Cz. indicate that it has a more planar conjugation keeping the HOMO-LUMO energy level at the lowest. The specific capacitance values of copolymers were lower than that of homopolymer. P[4In-3Cz] has the highest C_sp_ value among the series and this can be explained by the combination of oligomers formed from the 4In-3Cz comonomer that highly porous structure. In other words, P[4In-3Cz] possessed the most suitable structure for capacitive applications, while P[5In-3Cz] and P[6In-3Cz] were the most favorable candidates for electronic applications.

## Figures and Tables

**Table 1:** E_ox_ and I_p_ values of monomers.

Monomer	E_ox_, V	I_p,_ eV
In	1.14	5.54
Cz	1.24	5.64
[In+Cz]	1.16	5.56
4In-3Cz	1.06	5.46
5In-3Cz	1.19	5.59
6In-3Cz	1.00	5.40
7In-3Cz	1.09	5.49

**Table 2:** The conductivities, I_p_, E_g_, and C_sp_ of polymers.

Polymer	Conductivity, mS/cm	E_ox_, V	I_p_, eV	E_g_, eV	C_sp_, μF/cm^2^
PIn	0.3	0.41	4.81	2.70	1250
PCz	0.7	0.69	5.09	2.55	650
P[In-co-Cz]	0.2	0.55	4.95	2.87	840
P[4In-3Cz]	1.1	0.66	5.06	2.40	630
P[5In-3Cz]	1.0	0.52	4.92	2.16	530
P[6In-3Cz]	2.4	0.72	5.12	2.40	240
P[7In-3Cz]	0.1	0.78	5.18	3.21	110

**Table 3:** Impedance parameters of P[4In-3Cz] film obtained from R(Q(R(Q(RW)))) type equivalent circuit model.

Element	R_1_, ohm	CPE, Y_o_ S sec^n^	n	R_2_, ohm	CPE, Y_o_ S sec^n^	n	R_3_, ohm	Warburg, Y_o_ S sec^5^	C_sp_, _EIS_ μF/cm^2^	Chi-squared
Value	227.4	6.75 × 10^−7^	0.922	89.1	3.44 × 10^−7^	0.158	538.5	4.46 × 10^−7^	463	7.4 × 10^−4^

**Table 4:** The numbers and abbreviation of the name of possible dimers to be formed from the 4In-3Cz comonomer.

Dimer number	Abbreviation of the name of dimers
1	D[2,2′-(In-In)]_4In-3Cz_
2	D[2,3-(In-In)]_4In-3Cz_
3	D[3,3′-(In-In)]_4In-3Cz_
4	D[2,3-(In-Cz)]_4In-3Cz_
5	D[3,3-( In-Cz)]_4In-3Cz_
6	D[3,3′-(Cz-Cz)]_4In-3Cz_
7	D[2,3-( Cz-Cz)]_4In-3Cz_
8	D[2,2′-( Cz-Cz)]_4In-3Cz_

## References

[1] Morin JF Leclerc M Ades D Siove A Polycarbazoles: 25 Years of Progress Macromolecular Rapid Communication 2005 26 10 761 778 10.1002/marc.200500096

[2] Saraswathi R Gerard M Malhotra BD Characteristics of aqueous polycarbazole batteries Journal of Applied Polymer Science 1999 74 1 145 150 10.1002/(SICI)1097-4628(19991003)74:1<145::AID-APP18>3.0.CO;2-C

[3] Verghese MM Ram MK Vardhan H Malhotra BD Ashraf SM Electrochromic properties of polycarbazole films Polymer 1997 38 1625 10.1016/s0032-3861(96)00655-6

[4] Sivakkumar SR Angulakshmi N Saraswathi R Characterization of Poly (indole-5-carboxylic Acid) in Aqueous Rechargeable Cells Journal of Applied Polymer Science 2005 98 917

[5] Maarouf EB Billaud D Hannecart E Electrochemical cycling and electrochromic properties of polyindole Materials Research Bulletin 1994 29 6 637

[6] Pandey PC Prakash R Electrochemical Cycling and Electrochromic Properties of Polyindole Electrochemical Society 1998 145 3 999

[7] Pandey PC Prakash R Characterization of Electropolymerized Polyindole: Application in the Construction of a Solid-State, Ion-Selective Electrode Journal Electrochemical Society 1998 145 12 4103

[8] Machida K Hiraki R Takenouchi H Naoi K Redox capacitor properties of indole derivatives II. Electrochemical characteristics of substituted cyclic indole trimers Electrochemistry 2005 73 9 813 822

[9] Düdükçü M Udum YA Ergun Y Köleli F Electrodeposition of poly (4-methyl carbazole-3-carboxylic acid) on steel surfaces and corrosion protection of steel Journal of Applied Polymer Science 2009 111 3 1496 1500

[10] Somasundrum M Bannister JV Mediatorless electrocatalysis at a conducting polymer electrode: application to ascorbate and NADH measurement Journal Chemical Society Chemical Communication 1993 21 1629 1631

[11] Wan F Li L Wan X Xue G Modification of Polyindole by the incorporation of Pyrrole Unit Journal of Applied Polymer Science 2002 85 4 814 820

[12] Dhanalakshmi K Saraswathi R Electrochemical preparation and characterization of conducting copolymers: poly(pyrrole-co-indole) Journal Material Science 2001 36 17 4107

[13] Nie G Han X Zhang S Xu J Cai T Electrochemical copolymerization of indole and 3-methylthiophene Journal of Applied Polymer Science 2007 104 3129

[14] Xu JK Nie GM Zhang SS Han XJ Hou J Electrochemical copolymerization of indole and 3, 4-ethylenedioxythiophene Journal Material Science 2005 40 11 2867

[15] Saraç AS Serantoni M Tofail SAM Cunnane VJ Nanoscale Characterization of Carbazole–Indole Copolymers Modified Carbon Fiber Surfaces Journal of Nanoscience and Nanotechnology 2005 5 10 1677 16245527 10.1166/jnn.2005.406

[16] Kuo CW Hsieh TH Hsieh CK Liao JW Wu TY Electrosynthesis and Characterization of Four Electrochromic Polymers Based on Carbazole and Indole-6-Carboxylic Acid and Their Applications in High-Contrast Electrochromic Devices Journal of The Electrochemical Society 2014 161 14 D782 D790

[17] Kuo CW Wu TY Huang MW Electrochromic characterizations of copolymers based on 4,4-bis(N-carbazolyl)-1,1-biphenyl and indole-6-carboxylic acid and their applications in electrochromic devices Journal of the Taiwan Institute of Chemical Engineers 2016 68 481 488

[18] Hwang J Park J Kim YJ Ha YH Park CE Indolo[3,2-b]indole-Containing Donor–Acceptor Copolymers for High-Efficiency Organic Solar Cells Chemical Materials 2017 29 5 2135 2140 10.1021/acs.chemmater.6b04745

[19] Wadatkar NS Waghuley SA Electrical investigation on thiophene–indole conducting copolymers as synthesized through in situ chemical copolymerization route Polymer Bulletin 2020 77 4181 4196 10.1007/s00289-019-02967-w

[20] Katsoulidis AP Dyar SM Carmieli R Malliakas CD Michael R Copolymerization of terephthalaldehyde with pyrrole, indole and carbazole gives microporous POFs Journal of Material Chemistry A 2013 1 10465 10473

[21] Gobalasingham NS Ekiz S Pankow RM Livi F Bundgaard E Carbazole-based copolymers via direct arylation polymerization (DArP) for Suzuki-convergent polymer solar cell performance Polymer Chemistry 2017 8 30 4393 402

[22] Xiao H Deng Y Yuan J Gao P Zhao B Synthesis and Photovoltaic Properties of the Copolymers Based on Carbazole with Tetrathiophene Porphyrin Side Chains Linked by a Flexible Alkyl-interval Chinese Journal of Chemistry 2018 36 7 599 604 10.1002/cjoc.201700804

[23] Maertens F Toppet S Hoornaert GJ Compernolle F Incorporation of an indole-containing diarylbutylamine pharmacophore into furo [2, 3-a] carbazole ring systems Tetrahedron 2005 61 7 1715

[24] Bergman J Condensation of indole and formaldehyde in the presence of air and sensitizers: A facile synthesis of indolo[3.2-b] carbazole Tetrahedron 1970 26 13 3353

[25] Levesque I Bertrand PO Blouin N Leclerc M Zecchin S Synthesis and thermoelectric properties of polycarbazole, polyindolocarbazole, and polydiindolocarbazole derivatives Chemistry Materials 2007 19 2128

[26] Stille JK The Palladium-Catalyzed Cross-Coupling Reactions of Organotin Reagents with Organic Electrophiles New Synthetic Methods Angewandte Chemie International Edition in English 1986 25 508

[27] Yamamoto T Yamamoto A A novel type of polycondensation of polyhalogenated organic aromatic compounds producing thermostable polyphenylene type polymers promoted by nickel complexes Chemistry Letters 1977 6 4 353 356 10.1246/cl.1977.353

[28] Heywang G Jonas F Poly (alkylenedioxythiophene)-new, very stable conducting polymers Advanced Material 1992 4 2 116 118 10.1002/adma.19920040213

[29] Faid K Cloutier R Leclerc M Design of novel electroactive polybithiophene derivatives Macromolecules 1993 26 2501 2507

[30] Faid K Frechette M Ranger M Mazerolle L Levesque I Chromic Phenomena in Regioregular and Non-regioregular Polythiophene Derivatives Chemistry Materials 1995 7 1390

[31] Leclerc M Faid K Electrical and optical properties of processable polythiophene derivatives: structure-property relationships Advanced Materials 1997 9 1087

[32] McCullough RD The chemistry of conducting polythiophenes Advanced Materials 1998 10 93

[33] Rehahn M Schluter AD Wegner G Feast WJ Soluble poly (para-phenylene)s. 1. Extension of the Yamamoto synthesis to dibromobenzenes substituted with flexible side chains Polymer 1989 30 1054

[34] Pei Q Yang Y Efficient photoluminescence and electroluminescence from a soluble polyfluorene Journal of American Chemical Society 1996 118 7416

[35] Sotzing GA Reynolds JR Electrochromic conducting polymers via electrochemical polymerization of bis (2-(3, 4-ethylenedioxy) thienyl) monomers Chemistry Materials 1996 8 882

[36] Geissler U Hallensleben ML Rohde N Poly[arylene-alt-[bis(1-methylpyrrolylene)]s, 1. Synthesis and electrochemical polymerization of terarenes Macromolecular Chemistry and Physics 1996 197 2565 10.1002/macp.1996.021970819

[37] Sezer E VanHooren M Saraç AS Hallensleben ML Synthesis and electrochemical polymerization of ter-arenes based on N-ethyl carbazole and thiophene Journal of Polymer Science Part A Polymer Chemistry 1999 37 379 10.1002/(SICI)1099-0518(19990215)37:4<379::AID-POLA1>3.0.CO;2-I

[38] Sezer E Saraç AS Parlak EA Electrochemical synthesis of EDOT–ECZ–EDOT copolymer on carbon fiber micro-electrodes Journal of Applied Electrochemistry 2003 33 1233

[39] Saraç AS Sarıoğlan SÖ Dziomba T Sezer E Synthesis and electrocoating of indole–thiophene comonomer on carbon fiber microelectrode, and surface topography by AFM European Polymer Journal 2007 43 3392

[40] Cebeci FÇ Sezer E Saraç AS Synthesis and electrochemical characterization of bis (3,4-ethylene-dioxythiophene)-(4,4′-dinonyl-2,2′-bithiazole) comonomer Electrochimica Acta 2007 52 2158

[41] Sezer E Heinze J Voltammetric, EQCM, and in situ conductivity studies of 3, 6-bis (2-thienyl)-N-ethyl carbazole Electrochimica Acta 2006 51 3668

[42] Roncali J Conjugated poly (thiophenes): synthesis, functionalization, and applications Chemical Reviews 1992 92 711 738

[43] Bergman J Synthesis of 3-substituted indoles starting from isatin Acta Chimica Scandinavica 1971 25 4

[44] Benhida R Lecubin F Fourrey JL Castellanos LR Quintero L Synthesis of 6-allyl and 6-heteroarylindoles by palladium catalysed Stille cross-coupling reaction Tetrahedron Letters 1999 40 5701

[45] ASTM F42-93; Standard Test Methods for Conductivity Type of Extrinsic Semiconducting Materials Annual Book ASTM Standard 1997 Last Updated: Aug 16, 2017

[b46-turkjchem-46-5-1677] 46Keithley Application Note Series. Measuring the Resistivity and Determining the Conductivity Type of Semiconductor Materials Using a Four-Point Collinear Probe and the Model 6221 DC and AC Current 2005; Source Number 2615.

[47] Frisch MJ Trucks GW Schlegel HB Scuseria GE Robb MA Gaussian 03 Gaussian Inc Pittsburgh PA, Revision 2003 B:04

[48] Janietz S Bradley DDC Grell MM Giebeler C Inbasekaran M Electrochemical determination of the ionization potential and electron affinity of poly (9, 9-dioctylfluorene) Applied Physical Letter 1998 73 17 2453

[49] Vorotyntsev MA Zinovyeva VA Konev DV Mechanisms of Electropolymerization and Redox Activity: Fundamental Aspects Electropolymerization: Concepts, Materials and Applications Cosnier S Karyakin AA Weinheim, Germany Wiley-VCH 2010 27 50

[50] Nalwa HS Handbook of Organic Conductive Molecules and Polymer John Wiley New York 1997 V2 157

[51] Skompska M Szkurlat A The influence of the structural defects and microscopic aggregation of poly (3-alkylthiophenes) on electrochemical and optical properties of the polymer films Electrochimica Acta 2001 46 4007

[52] Jiang X Harima Y Yamashita K Tada Y Ohshita J Doping-induced change of carrier mobilities in poly (3-hexylthiophene) films with different stacking structures Chemistry and Physics Letters 2002 364 616

[53] Jiang X Patil R Harima Y Ohsita J Kunai A Influences of self-assembled structure on mobilities of charge carriers in π-conjugated polymers Journal Physics Chemisty B 2005 109 221 10.1021/jp0460994 16851008

[54] Visy C Lukkari J Kankare J A thermodynamic approach to the interpretation of anodic and cathodic doping of poly (3-methylthiophene) Journal Electroanalytical Chemistry 1991 319 85

[55] Girija TC Sangaranarayanan MV Investigation of polyaniline-coated stainless-steel electrodes for electrochemical supercapacitors Synthetic Metals 2006 156 2–4 244 250

[56] Waltman RJ Diaz AF Bargon J Substituent Effects in the Electropolymerization of Aromatic Heterocyclic Compound Journal of Physical Chemistry 1984 88 19 4343 4346

[57] Jackowska K Kudelski A Bukowska J Spectroelectrochemical and EPR determination of the number of electrons transferred in redox process in electroactive polymers polyindole films Electrochimica Acta 1994 39 10 1365 1368

[58] Talbi H Monard G Loos M Billaud D Theoretical study of indole polymerization Journal of Molecular Structure: Theochem 1998 434 129 134

[59] Talbi H Monard G Loos M Billaud D Theoretical investigation of the monomer reactivity in polyindole derivatives Synthetic Metals 1999 101 115 116

[60] Dubnikova F Lifshitz A Isomerization of indole. Quantum chemical calculations and kinetic modelling Journal Physical Chemistry A 2001 105 3605 3614

[61] Yurtsever M Yurtsever E A DFT study of polymerization mechanism of indole Polymer 2002 43 6019 6025

[62] Saraç AS Sezer E Ustamehmetoğlu B Oxidative polymerization of N-substituted carbazoles Polymer Advanced Technologies 1997 8 9 556 562

[63] Waltman RJ Bargon J Electrically conducting polymers: a review of the electropolymerization reaction, of the effects of chemical structure on polymer film properties, and of applications Canadian Journal Chemistry 1986 64 76

[64] Skotheim TA Handbook of Conducting Polymers; Marcel Dekker Inc New York 1986

[65] Agata T Cybulski H Chmielewski MJ Bukowskaa J Skompska M Electrochemical and spectroscopic characterization of poly(1,8-diaminocarbazole): Part I. Electropolymerization and determination of the polymer structure by FTIR studies and DFT calculations Electrochimica Acta 2009 54 4743

